# Severe Pneumonia Caused by Infection With *Tropheryma whipplei* Complicated With *Acinetobacter baumannii* Infection: A Case Report Involving a Young Woman

**DOI:** 10.3389/fpubh.2021.729595

**Published:** 2021-10-25

**Authors:** Sheng Wang, Demeng Xia, Jianghong Wu, Dandan Jia, Lei Li, Shuogui Xu

**Affiliations:** ^1^Department of Emergency, Changhai Hospital, Naval Medical University, Shanghai, China; ^2^Health Clinic, Liberation Army Unit 91666, Zhoushan, China

**Keywords:** *Tropheryma whipplei*, severe pneumonia, *Acinetobacter baumannii*, bronchoalveolar lavage, metagenomics next-generation sequencing

## Abstract

Whipple's disease is a very rare systemic infectious disease, and very few cases have been reported. However, it can be fatal if not diagnosed and treated appropriately. The major clinical manifestations of this disease are usually digestive and nervous system symptoms. The majority of patients are male and between 40 and 50 years old. Although respiratory symptoms of this disease have rarely been reported, they pose a serious threat to the lives of the patients, especially when they progress to severe pneumonia. During admission to the hospital, *Acinetobacter baumannii* infection makes treatment more difficult. While most patients are middle-aged men, more attention should be given to the diagnosis and treatment of affected young women. To our knowledge, the case presented in the study is the first case of *Tropheryma whipplei* infection that resulted in severe pneumonia and was complicated by *A. baumannii* infection during treatment. We hope that our study can serve as a reference for the diagnosis and treatment of related cases in the future.

## Background

*Tropheryma whipplei* infection was first discovered in 1907; it was identified as a rare chronic infectious disease with an annual incidence of 3 in one million population, and a male to female patient ratio of 8 to 1 (1, 2). The most obvious symptom of the prodromal phase of the disease is joint pain, while in the stable phase, it mainly manifests as abdominal pain with diarrhea and weight loss. Later, it develops into myocarditis, pericarditis and inflammation of the nervous system. To date, there have been reports about cases of this disease damaging the eyes, spine and skin (3–5). Because of the involvement of multiple systems, the clinical manifestations are relatively complex, so it is extremely easy to miss the diagnosis and arrive at a misdiagnosis, resulting in inappropriate treatment decisions, resulting in adverse outcomes. Here, we report a rare case of *T. whipplei* infection that led to “severe pneumonia,” which was the primary complaint. During hospitalization, the patient became infected with *Acinetobacter baumannii*. After a prompt diagnosis and appropriate treatment, the patient recovered and was discharged. Taking into account the background of the coronavirus disease 2019 (COVID-19) pandemic and the uniqueness of this disease, we would like to share our team's experience in the diagnosis and treatment of this rare disease with clinicians and other readers.

## Case Presentation

**Figure 1:**
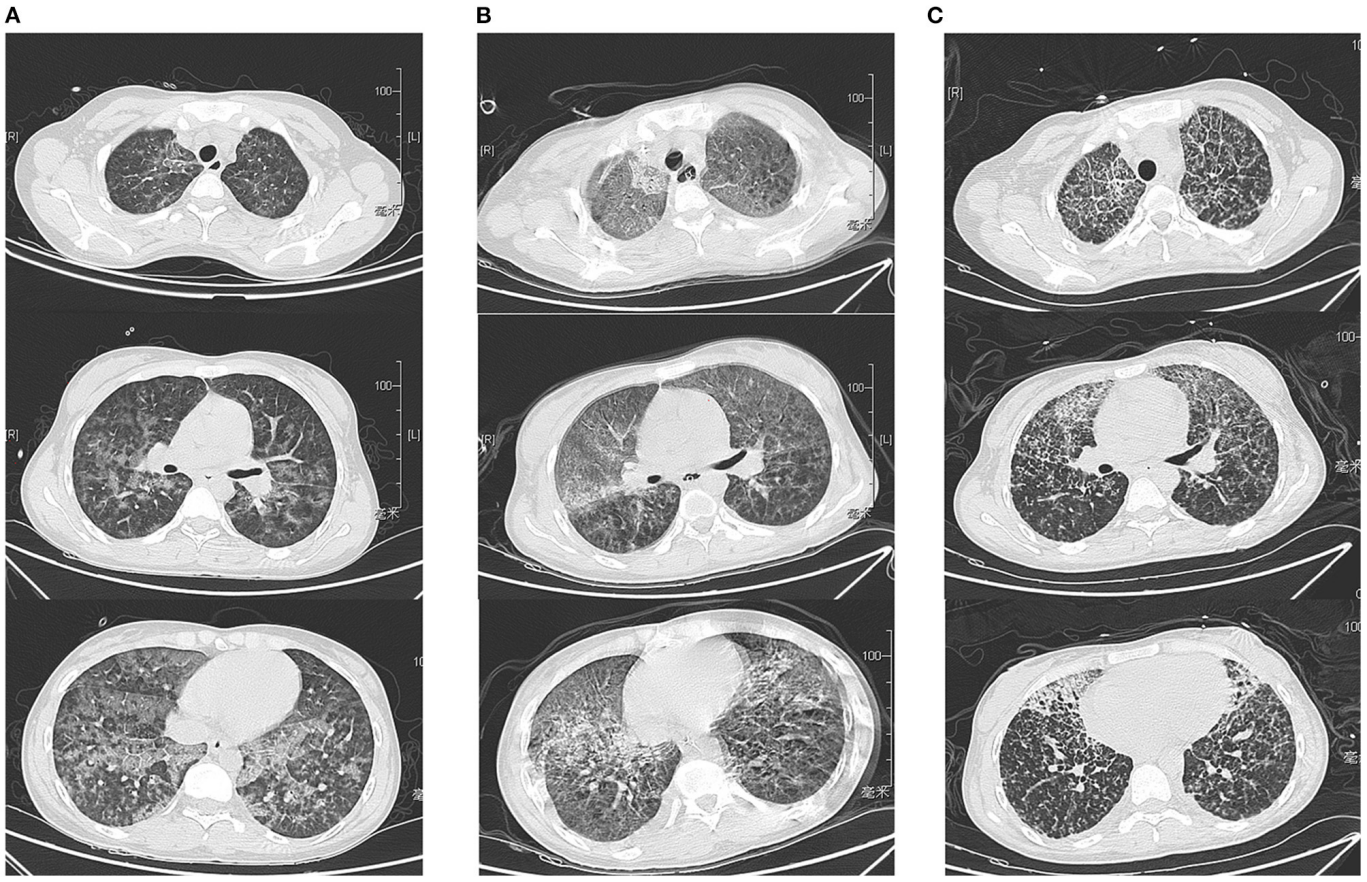
Chest CT results on day 1, day 7, and day 14 after admission. **(A)** Chest CT findings on the first day of admission revealed diffuse pneumonitis throughout the lungs. **(B)** The patient's chest CT on day 7 showed a worsening of the infection (due to the severity of the disease, the patient could not hold her breath well, so the images were not clear). **(C)** On the 14th day of admission, the patient's chest CT results showed slight improvement after treatment.

A 23-year-old woman was admitted with chest distress and difficulty breathing. The patient said that 1 week prior, she had symptoms of fever, bloody stools and headache, and oral antibiotics did not relieve the symptoms. On admission, the vital signs observed during the physical examination were as follows: body temperature, 39.6 °C; blood pressure, 82/41 mmHg; pulse, 116 beats/min; and saturated oxygen in arterial blood, 91%. Chest CT results showed that extensive blurring in both lungs, and diffuse inflammation of both lungs was considered (Figure 1A). In the context of the COVID-19 pandemic, we first considered the possibility that the patient might have COVID-19; however, only 2 h later, the result of a nucleic acid test of a throat swab was negative, which helped us rule out COVID-19. At the same time, an emergency blood test showed that her white blood cell count was 2.48 × 10^9^/L, with a neutrophil percentage of 90.3% and a lymphocytes percentage of only 3.1%. Given the large disparity in the percentages of lymphocytes and neutrophils, we were more inclined to suspect infections with microorganisms other than viruses.

Other examination results were as follows: the levels of procalcitonin (PCT) was 24.55 ng/mL, respectively. According to the above examination results, the patient had a serious infection. Given the patient's high PCT level and clinical symptoms, we considered a bacterial infection, but the low white blood cell and neutrophil levels made it impossible for us to rule out other infections. We have obtained more information about the patient's situation and found that she had lost 5 kg in 3 months and experienced joint pain, but she did not pay attention to these symptoms at the time. Therefore, we had reason to suspect she may be infected with HIV. However, the examination results showed that the HIV antibody level was within normal limit, which forced us to reconsider. Due to the possibility of a fungal or bacterial infection and the need to gain control over that infection immediately, the broad-spectrum antimicrobial drugs imipenem and fluconazole were administered.

On the second day of admission, the blood gas analysis results showed that the patient's arterial oxygen partial pressure had decreased to 50 mmHg, and her partial pressure of carbon dioxide was 75 mmHg. The patient was diagnosed with type I respiratory failure. At the same time, the patient's oxygen saturation level decreased from 90 to 70%. Therefore, endotracheal intubation with mechanical ventilation was performed, and the synchronized intermittent mandatory ventilation (SIMV) was selected as the ventilator mode. The tidal volume was 350 ml (her current weight was 50 kg), the positive end-expiratory pressure (PEEP) was 3 cmH_2_O, the oxygen concentration was 35%, and the frequency was 12 times/min. The patient's oxygen saturation was maintained at 100%. At that point, the patient had met the diagnostic standard for severe pneumonia. In addition, the patient's liver function and renal function were impaired to varying degrees: her creatinine level was 293 μmol/L, her alanine aminotransferase level was 66 U/L, her aspartate aminotransferase level was 121 U/L, her platelet count was significantly reduced to 37 × 10^9^/L, and her urine volume was also reduced to < 0.5 ml/kg/h. However, all the above indexes had been normal before disease onset. The patient was given conventional treatments such as inhaled oxygen, fluid replacement, antitussive treatment, diuresis and liver protection, and antibiotics continued to be administered. Her blood pressure remained at approximately 110/70 mmHg on dopamine.

**Figure 2:**
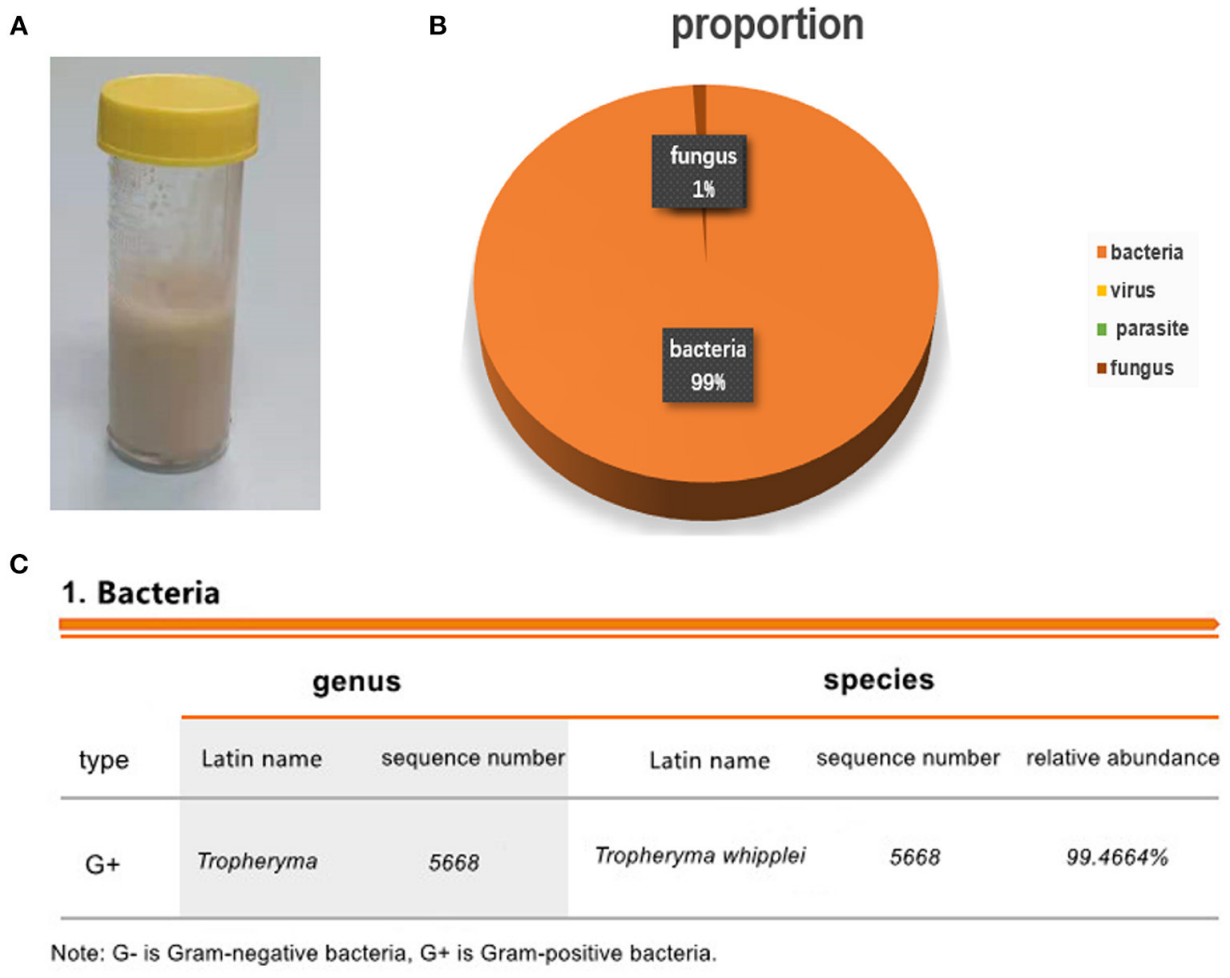
Results of the next-generation sequencing of bronchoalveolar lavage fluid. **(A)** Bronchoalveolar lavage fluid. **(B)** The results showed that the relative abundance of *Tropheryma whipplei* was 99.4664%. The number of sequences was 5668. **(C)** The proportions of bacteria, viruses, parasites and fungi.

**Figure 3:**
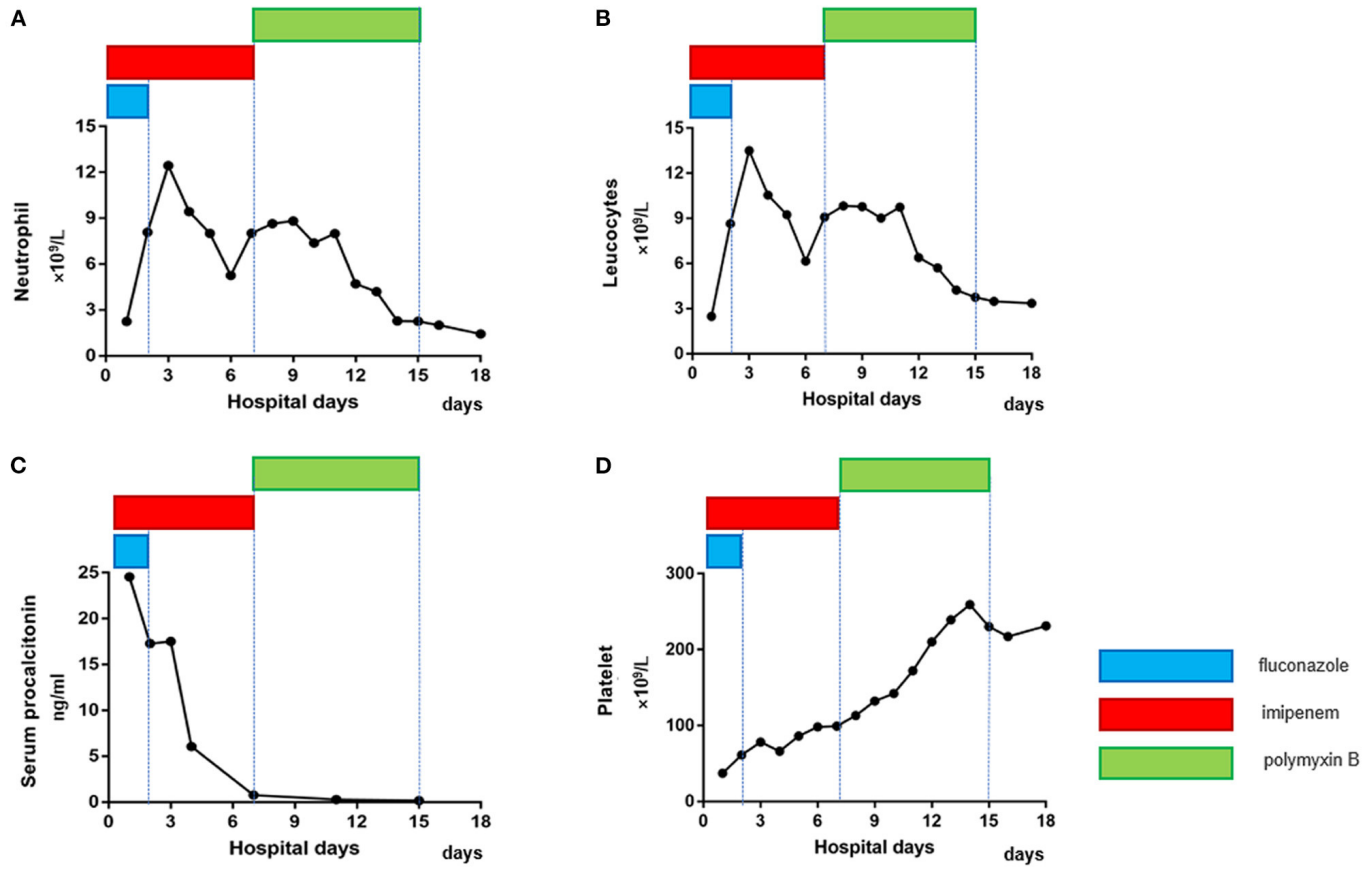
Dynamic changes in infection indexes and platelet counts in the patient after admission. **(A, B)** The neutrophil and leucocyte counts decreased significantly 7 days after admission. **(C)** The serum procalcitonin level continued to decline. **(D)** The platelet count continued to increase. Eventually, the patient's indicators returned to normal (different colors represent different medications, and the length of the lines represents the duration of treatment with the medication).

**Figure 4:**
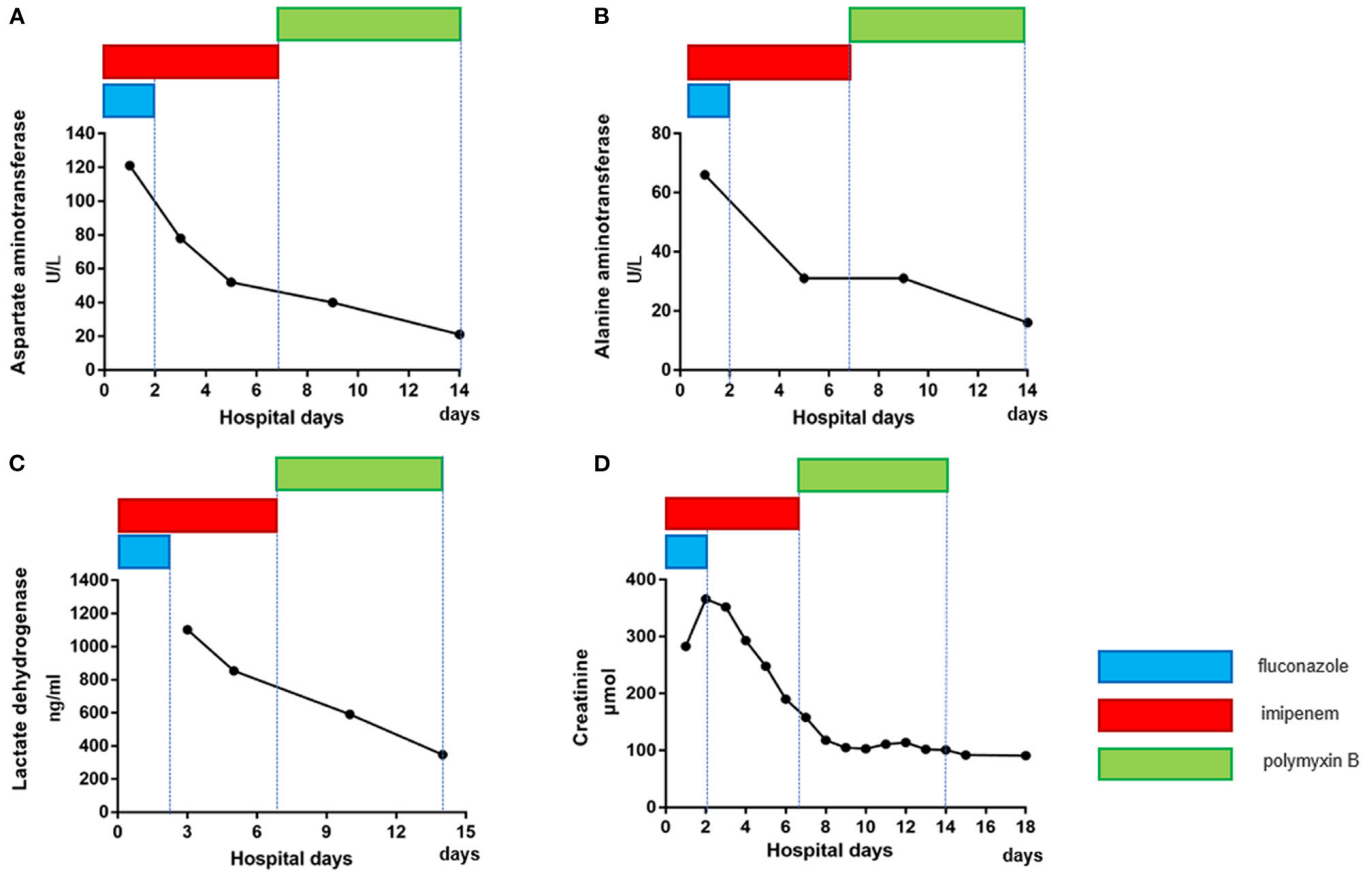
Dynamic changes in liver and kidney function in the patient after admission. After appropriate treatment, the aspartate aminotransferase **(A)**, alanine aminotransferase **(B)**, lactate dehydrogenase **(C)** and creatinine **(D)** levels of the patient gradually returned to normal (different colors represent different medications, and the length of the lines represents the duration of treatment with the medication).

Considering that the results of blood and sputum cultures take time to be returned and substantial wet rales could be heard in both lungs on auscultation, we performed fibreoptic bronchoscopy to enable us to identify the pathogen and provide the appropriate treatment as soon as possible. A large amount of white mucous was found in the airway during the procedure, and bronchoalveolar lavage fluid (BALF) (Figure 2A) was obtained for next-generation sequencing (NGS). The gene sequencing results for the pathogenic microorganisms were as follows: the relative abundance of *T. whipplei* was *99.4664%*, and the sequence number was *5,668* (fungi, parasites, chlamydia, mycoplasma, mycobacterium and other types of bacteria were not detected) (Figures 2B,C). After identifying the pathogen as a gram-positive bacterium, fluconazole was discontinued, and imipenem was maintained. In the following week, we re-examined the patient's laboratory parameters (Figures 3, 4), and the patient's overall performance had slightly improved.

However, on the seventh day of admission, her PCT value decreased significantly. However, there was a slight rebound in her white blood cell count, and the patient's body temperature also remained at approximately 38 degrees Celsius and did not return to normal. Even more striking was the fact that her chest CT findings worsened (Figure 1B). At the same time, the drug sensitivity results of the bacterial culture were obtained. Unfortunately, the results of the bacterial culture of patients showed that the main pathogenic bacterium had changed from *T. whipplei* to carbapenem-resistant *A. baumannii*, which was resistant to all tested antibiotics.

After careful consideration, treatment with the previous antibiotics was stopped, and we made the decision to treat the patient empirically by adding polymyxin B to our treatment plan, given that there were no other drugs available because the *A. baumannii* was resistant to all tested antibiotics. Fortunately, the patient's condition gradually returned to normal. Her liver function, kidney function (Figure 4), platelet count and infection indexes improved significantly (Figure 3), although her chest CT scans showed that the infection in the lungs had improved only slightly after treatment (Figure 1C). Twelve days after admission, the patient's respiratory function had improved, and she was weaned from the ventilator and extubated. All antibiotics were discontinued on the 15th day after admission, and the patient was able to get out of bed and perform simple activities 16 days after admission. She was discharged 18 days after admission.

## Discussion

Severe pneumonia is a common disease encountered in clinical practice, but its diagnosis and treatment are often challenging, especially when pneumonia is cause by infections with rare or unknown pathogens. The patient we admitted was treated for symptoms such as hypotension, high fever, dyspnoea and low white blood cell count and was initially diagnosed with severe pneumonia. After the test results were obtained, viral infections, including infection with HIV, were ruled out. Considering that bacterial cultures take a long time, we obtained bronchoalveolar lavage fluid and performed next-generation sequencing. At present, nearly all infectious agents contain DNA or RNA genomes, making sequencing an attractive approach to pathogen detection. Next-generation sequencing has emerged as means of detecting and taxonomically characterizing microorganisms in clinical samples from patients (6). At present, for patients with severe infections and unknown causative pathogen, early next-generation sequencing is necessary to guide the selection of a reasonable treatment plan as soon as possible. The rapid, sensitive results of next-generation sequencing make it far superior to microbial culture. In addition, studies have shown that next-generation sequencing has apparent advantages for the detection of viruses and co-infections, *at present, it has been fully used in the diagnosis of rare bacteria* (7). Finally, in this case, infection with *T. whipplei* was confirmed in only 1 day.

*Tropheryma whipplei* is a gram-positive actinomycete that is a symbiotic bacterium and is widespread in the external environment. It is unlikely to cause disease in normal people, although it can cause infections in patients with partial immune deficiency. *Tropheryma whipplei* causes a chronic infectious disease known as Whipple's disease, which was first described in 1907 by G.H. Whipple and has an annual incidence of 3 in one million population (1). Classic Whipple's disease mainly involves the digestive system and is characterized by a tetrad of symptoms: arthralgia, weight loss, diarrhea, and abdominal pain. Gastrointestinal findings are the most common clinical manifestations that are identified in approximately 90–95% of patients (8). Due to the large proportion of cases involving the digestive system, there are now relatively mature diagnostic and therapeutic measures. A definite diagnosis of classic Whipple's disease is generally made by observing the massive infiltration of foamy macrophages containing dense PAS-positive granules in the lamina propria on histopathological examination of the biopsy material obtained from the small intestine (9). However, cases involving the respiratory system are rare. Existing studies have shown that respiratory infection is reported in only 13–14% of cases (10, 11) and usually involve pleural effusion and/or pulmonary hypertension (8). However, our patient presented with dyspnoea, chest distress and cough as the main symptoms, which is very rare in the literature. Another concern was the age and sex of the patient. According to an Italian study (1), the disease is more likely to occur in middle-aged white males, and the incidence in females is relatively low, with female patients accounting for only 30%. However, a study in the United States (12) found that the sex ratio is changing and that currently there is no significant difference in incidence between men and women. Nevertheless, this requires us to pay more attention to the atypical manifestations of the disease and the diversity of the patient population in clinical practice to improve our diagnostic success and treatment of patients.

To date, the treatment of Whipple's disease with pulmonary symptoms as the main complaints is not yet mature and has not been standardized. In our case, based on the next-generation sequencing results, we changed the treatment regimen in a timely manner, and the patient's condition showed significant improvement. On the 8^th^ day, however, the patient's condition changed again, and the bacterial culture showed *A. baumannii*. This is a gram-negative 0 bacteria common in nosocomial infections, especially in intensive care units (ICUs) and immunocompromised patients with central venous catheters; it is often multi-drug resistant (MDR) and usually infects the respiratory tract, especially in those on mechanical ventilation (13). Antibiotics that are usually effective against *A. baumannii* infections include polymyxins E and B, sulbactam, piperacillin/tazobactam, tigecycline, aminoglycosides and carbapenems. The patient in our case had nearly all of the risk factors for infection with *A. baumannii*. Unfortunately, in this case, the *A. baumannii* was not sensitive to *any of the tested antibiotics*. We made the decision to treat the patient empirically by adding polymyxin B to our treatment plan. To our surprise, the patient's white blood cell and neutrophil counts returned to normal after a few days, and her lung CT showed improvement. Finally, all antibiotics were discontinued on the 15th day of admission, and the patient was discharged on the 18th day. This also suggests that pathogens that show resistance to antibiotics can sometimes still be treated with empiric therapy. Of course, we cannot deny that early drug sensitivity testing is essential for infection control in cases of serious infections. In addition, we believe that the patient's recovery was also due to her young age, which was conducive to the recovery of her physiological function.

## Conclusions

To our knowledge, this is the first case of severe pneumonia caused by infection with *T. whipplei* complicated with *A. baumannii* infection. The atypical natures of both the affected system and the patient deserve our attention. In terms of diagnosis, next-generation sequencing is relatively faster, supporting the early diagnosis of serious infections. As one of six main pathogens involved in nosocomial infections, *A. baumannii* also poses a clinical challenge; therefore, the timely performance of drug sensitivity tests and the selection of appropriate antibiotics are important. We hope that our report can serve as a reference for the diagnosis and treatment of this disease, and more clinical data are expected to be published to provide more support.

## Data Availability Statement

The original contributions presented in the study are included in the article/supplementary material, further inquiries can be directed to the corresponding authors.

## Ethics Statement

Written informed consent was obtained from the individual(s) for the publication of any potentially identifiable images or data included in this article.

## Author Contributions

SW and DX collected and analyzed the data and wrote the manuscript. JW, DJ, LL, and SX designed the study and revised the manuscript. All authors have read and approved the final manuscript.

## Conflict of Interest

The authors declare that the research was conducted in the absence of any commercial or financial relationships that could be construed as a potential conflict of interest.

## Publisher's Note

All claims expressed in this article are solely those of the authors and do not necessarily represent those of their affiliated organizations, or those of the publisher, the editors and the reviewers. Any product that may be evaluated in this article, or claim that may be made by its manufacturer, is not guaranteed or endorsed by the publisher.
